# The Prognostic Value of *BRAF* Mutation in Colorectal Cancer and Melanoma: A Systematic Review and Meta-Analysis

**DOI:** 10.1371/journal.pone.0047054

**Published:** 2012-10-09

**Authors:** Gholamreza Safaee Ardekani, Seyed Mehdi Jafarnejad, Larry Tan, Ardavan Saeedi, Gang Li

**Affiliations:** 1 Department of Dermatology and Skin Science, Jack Bell Research Centre, Vancouver Coastal Health Research Institute, University of British Columbia, Vancouver, British Columbia, Canada; 2 Department of Statistics, University of British Columbia, Vancouver, British Columbia, Canada; Ohio State University Medical Center, United States of America

## Abstract

**Background:**

Mutation of *BRAF* is a predominant event in cancers with poor prognosis such as melanoma and colorectal cancer. *BRAF* mutation leads to a constitutive activation of mitogen activated protein kinase pathway which is essential for cell proliferation and tumor progression. Despite tremendous efforts made to target BRAF for cancer treatment, the correlation between *BRAF* mutation and patient survival is still a matter of controversy.

**Methods/Principal Findings:**

Clinical studies on the correlation between *BRAF* mutation and patient survival were retrieved from MEDLINE and EMBASE databases between June 2002 and December 2011. One hundred twenty relevant full text studies were categorized based on study design and cancer type. Publication bias was evaluated for each category and pooled hazard ratio (HR) with 95% confidence interval (CI) was calculated using random or fixed effect meta-analysis based on the percentage of heterogeneity. Twenty six studies on colorectal cancer (11,773 patients) and four studies on melanoma (674 patients) were included in our final meta-analysis. The average prevalence of *BRAF* mutation was 9.6% in colorectal cancer, and 47.8% in melanoma reports. We found that *BRAF* mutation increases the risk of mortality in colorectal cancer patients for more than two times; HR = 2.25 (95% CI, 1.82–2.83). In addition, we revealed that *BRAF* mutation also increases the risk of mortality in melanoma patients by 1.7 times (95% CI, 1.37–2.12).

**Conclusions:**

We revealed that *BRAF* mutation is an absolute risk factor for patient survival in colorectal cancer and melanoma.

## Introduction

The mitogen activated protein kinase (MAPK) pathway is one of the most crucial pathways in regulation of cancer cell proliferation and survival [1]. Constitutive activation of the MAPK pathway in cancers has been frequently observed in various malignancies which is usually due to activating mutations in upstream factors such as RAS and RAF [2]. Accordingly, mutations in *BRAF* are reported in up to 70% of cancer cell lines [3] and they are highly prevalent in most common cancers with poor prognosis such as malignant melanoma [3], [4]. Mutations in *BRAF* have been reported in up to 60% of melanoma cases, between 40 to 70% of thyroid carcinomas, and up to 18% of colorectal cancers [3], [5].

So far, over 50 distinct mutations have been identified in the *BRAF* gene, which are present either in the glycine-rich P-loop of the N lobe or the activating segment in the exon 15 region [6]. Most of these mutations increase BRAF activity by 1.5 to 700 folds depending on the type of the mutation [6]. Of all *BRAF* activating mutations, a transitional mutation in nucleotide 1799 (T-A), also known as *BRAF*-V600E, is the most common change. In fact, this single mutation dramatically increases BRAF activity and accounts for more than 80% of all reported *BRAF* mutations in tumors [3], [6]. This point mutation results in a valine to glutamic acid substitution that exposes the active site (normally sealed in a hydrophobic pouch) and implicates the constitutive activation of BRAF. As a result, malignant cells with V600E mutation proliferate in a growth factor-independent manner in culture as well as in tumors in animal models [7]. In addition, it has been demonstrated that *BRAF* mutation is highly involved in main steps of cancer development and progression [8]. Together, these reports nominate the *BRAF*-V600E mutation as a very promising therapeutic target in *BRAF* mutated cancers. So far, BRAF inhibitor PLX4032 is one of the only few promising treatments for malignant melanoma approved by the US Food and Drug Administration.

Although there are multiple reports on the correlation of *BRAF* mutation with a variety of cancer progression steps, the correlation between *BRAF* mutation and cancer patient survival is still a matter of controversy in different reports [9]–[15]. In this study, we used systematic review and meta-analysis as the most reliable approach to investigate whether *BRAF-*V600E mutation is associated with patient outcome. A pool of studies published between 2002 and 2011 on the association between *BRAF*-V600E mutation and patient survival in colorectal cancer, malignant melanoma and papillary thyroid carcinoma were reviewed and analyzed for this study. We found that *BRAF* mutation increases the risk of mortality in colorectal cancer patients by more than two-fold. In addition, we revealed that *BRAF*-*V600E* mutation also increases the risk of mortality in melanoma patients by 1.7 times, while its effect on papillary thyroid carcinoma still requires further investigation.

## Methods

### Search Strategy and Selection Criteria

We conducted a comprehensive search of medical literature on studies evaluating the effect of *BRAF*-V600E mutation on cancer patient survival. We searched MEDLINE and EMBASE using the terms “*BRAF*”, “*BRAF* mutation”, “*BRAF* V600E”, “cancer”, “patient survival”, “colorectal cancer”, “melanoma”, and “papillary thyroid carcinoma” in different combinations from June 2002 to December 2011. We initially narrowed our search based on research title followed by abstract and finally full texts were reviewed if they were categorized as relevant reports. We did not restrict the language in our research. All of the references from review papers and original reports were checked for further relevant studies in the systematic review.

Studies were excluded if contained no clinicopathologic data, survival analysis, or no comparison between wild type and mutant *BRAF*. In addition, studies which only reported a progression free survival as well as *in vitro* and animal reports were also excluded. For more information in detail please refer to PRISMA checklist (Table S1).

### Data Extraction and Study Assessment

Two independent reviewers (GSA and LT) reviewed each full text report for eligibility and extracted required data. For each study the data on the number of patients in each group, mean survival time, hazard ratio and mean progression free survival time for randomized controlled trials (RCT), cancer type and study design were obtained and a consensus was achieved on all items. In the cases of incomplete required information, authors were contacted for additional information which was added as best as possible. Duplication of data was avoided by matching the author’s name and the name of the research centers.

### Statistical Analysis

We started summarizing the effect of *BRAF*-V600E mutation on patient survival separately based on study design RCT versus cohort and cancer type. We evaluated the publication bias using funnel plot analysis. We also assessed the heterogeneity of the studies using chi-square test of heterogeneity and I^2^ measure of inconsistency. Significant heterogeneity was defined as a Chi-square test *P* value of <0.10 or as an I^2^ measure >50%. Estimated hazard ratio (HR) was calculated using odds ratio and confidence interval in studies where HR was not available. In the absence of heterogeneity HRs and CIs were calculated according to a fixed model [16] which assumes that results across studies differ only by sampling error. In those studies where only the survival curve was available with no other detailed information, survival rates were extracted over multiple time periods in order to reconstruct HR and its variance with the assumption that patient censor rate was constant during study follow-up. This method has been described previously by Parmar *et al.* [17] to extract summary statistics for meta-analysis. A HR>1 was considered as a risk factor for worse survival in patient with positive *BRAF* mutation. In the end we used a log hazard ratio in the pooled data for the final analysis using R software (2011, The R Foundation for Statistical Computing). The impact of *BRAF* mutation on patient survival was considered statistically significant if 95% confidence interval for individual or overall log HR did not overlap zero.

## Results

### Number of Studies

**Figure 1:**
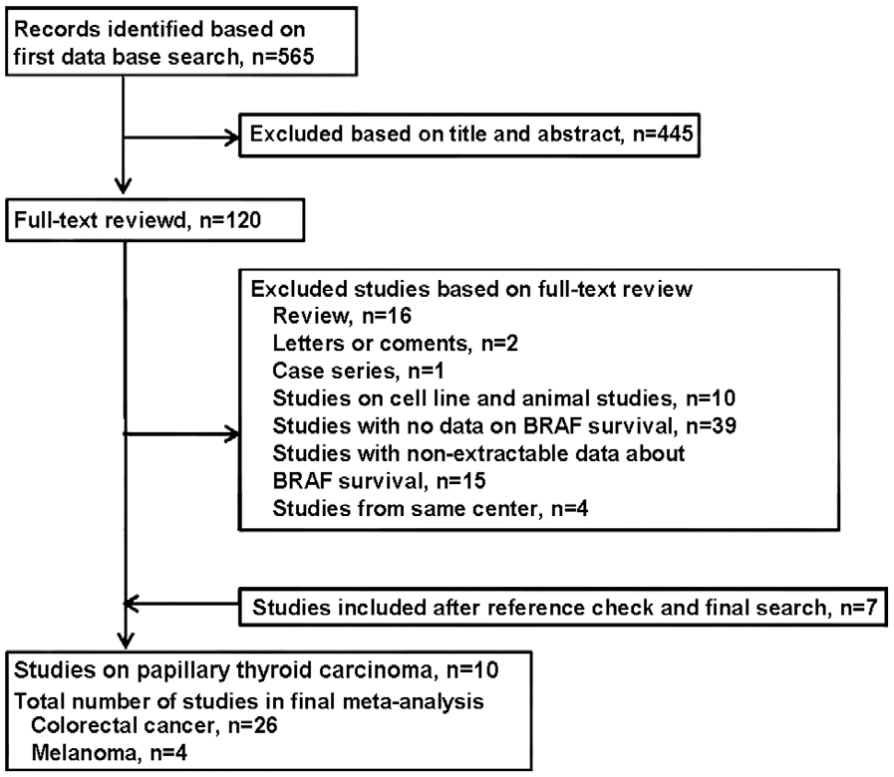
Flow diagram demonstrates the study selection process.

A total of 565 studies were retrieved from our electronic search. Of these, 120 abstracts were considered relevant and full texts were reviewed in detail. By the end of the review 26 studies on colorectal cancer (5 RCTs and 21 cohorts; 11,773 patients) met our inclusion criteria for meta-analysis. In addition, four studies on melanoma (1 RCT and 3 cohorts; 674 patients) including one study published at the time of statistical analysis [18] were incorporated in our final meta-analysis (Figure 1). Please also refer to complete PRISMA flow diagram (Figure S1) for more information. We were able to extract the overall survival information from two studies on papillary thyroid carcinoma [19], [20]. However, we did not perform meta-analysis on papillary thyroid carcinoma subject due to the small number of studies (Table 1). The funnel plot for colorectal cancer but not for melanoma studies showed a publication bias in our collected data.

**Table 1:** Summary of studies that evaluated the impact of *BRAF* mutation on overall patient survival in colorectal cancer and melanoma.

	Country	Studydesign	Number of patients				Overall survival		Hazard ratio
			Overall	*BRAF* subgroup	*BRAF* WT	*BRAF* mutant	*BRAF* mutant	*BRAF*WT	
**COLORECTAL CANCER**									
**Barault L ** [**11**]	France	Cohort	582	582	506	76(13.1%)			1.2(0.55–2.61)
**De Roock W ** [**55**]	Belgium	Cohort	886	761	725	36(4.7%)	26	54	2.93(1.85–4.65)
**Farina-Sarasqueta A ** [**45**]	Netherland	Cohort	258	203	165	38(18.7)			2.22(0.87–3.57)
**Ferracin M ** [**56**]	Italy	Cohort	93	79	72	7(8.9%)			3.37
**French AJ ** [**12**]	USA	Cohort	533	490	413	77(15.7%)	71	68	1.2(0.8–1.8)
**Laurent-Puig P ** [**57**]	France	Cohort	173	115	110	5(4.3%)	14.4	17.9	
**Liao W ** [**58**]	China	Cohort	61	61	58	3(4.9%)	9	11	2.016(0.61–6.58)
**Liou JM ** [**59**]	Taiwan	Cohort	314	314	302	12(3.8%)			3.91(1.31–11.66)
**Loupakis F ** [**48**]	Italy	Cohort	138	87	74	13(14.9%)	4.1	13.1	1.96(0.48–3.44)
**Maestro ML ** [**60**]	Spain	Cohort	351	324	312	12(3.7%)	41	68	1.62(0.50–5.21)
**Maughan TS ** [**61**]	UK	RCT	1630	1291	1189	102(7.9%)	8.8	14.4	
**Ogino S ** [**62**]	USA	Cohort	649	631	526	105(16.6%)			1.97(1.13–3.42)
**Park JH ** [**63**]	Korea	Cohort	75	71	66	5(7%)	2.46	7.53	3.06
**Price TJ ** [**49**]	Australia	Cohort	471	315	282	33(10.5%)	8.6	20.8	2.04(1.20–2.87)
**Richman SD ** [**34**]	UK	RCT	2135	692	638	54(7.8%)			1.82(1.36–2.43)
**Roth AD ** [**41**]	Switzerland	RCT	1404	1307	1204	103(7.9%)			1.59(0.65–3.91)
**Samowitz WS ** [**64**]	USA	Cohort	763	763	723	40(5.2%)			4.23(1.65–10.84)
**Saridaki Z ** [**65**]	Greece	Cohort	112	112	104	8(7.1%)	4.3	15.1	3.6(1.7–7.5)
**Shaukat A ** [**66**]	USA	Cohort	194	165	129	36(21.8%)			1.95(1.18–3.20)
**Souglakos J ** [**67**]	Greece/USA	Cohort	168	168	155	13(7.7%)	10.9	40.5	4.5(2.4–8.4)
**Tie J ** [**68**]	Australia	Cohort	525	525	473	52(9.9%)	2.8	13.5	2.48(1.31–4.72)
**Tol J ** [**69**]	Netherland	RCT	559	518	473	45(8.7%)	12.9	24.5	3.2
**Tran B ** [**70**]	Australia/USA	Cohort	524	524	467	57(10.9%)	10.4	34.7	11.11(6.27–19.17)
**Van Cutsem E ** [**9**]	Belgium	RCT	999	625	566	59(9.4%)	14.1	25.1	1.1(0.42–1.78)
**Yokota T ** [**71**]	Japan	Cohort	319	229	214	15(6.5%)	11	40.6	4.23(1.76–10.2)
**Zlobec I ** [**13**] **(Left side)**	Switzerland	Cohort	404	242	223	19(7.9%)			0.53(0.3–1.2)
**Zlobec I ** [**13**] **(Right side)**	Switzerland	Cohort	404	127	102	25 (19.7%)			2.82(1.5–5.5)
**MELANOMA**									
**Kumar R ** [**72**]	Finland	Cohort	38	38	12	26 (68.4%)			2.16(1.02–4.59)
**Long GV ** [**73**]	Australia	Cohort	197	197	102	95 (48.2%)	11.1	46.1	
**Si L ** [**18**]	China	Cohort	432	395	297	98 (24.8%)	33	53	1.54(1.11–2.12)
**von Moos R ** [**74**]	Switzerland	RCT	62	44	22	22 (50.0%)	9.2	12	

### Impact of *BRAF-V600e* Mutation on Colorectal Cancer Patient Survival

**Figure 2:**
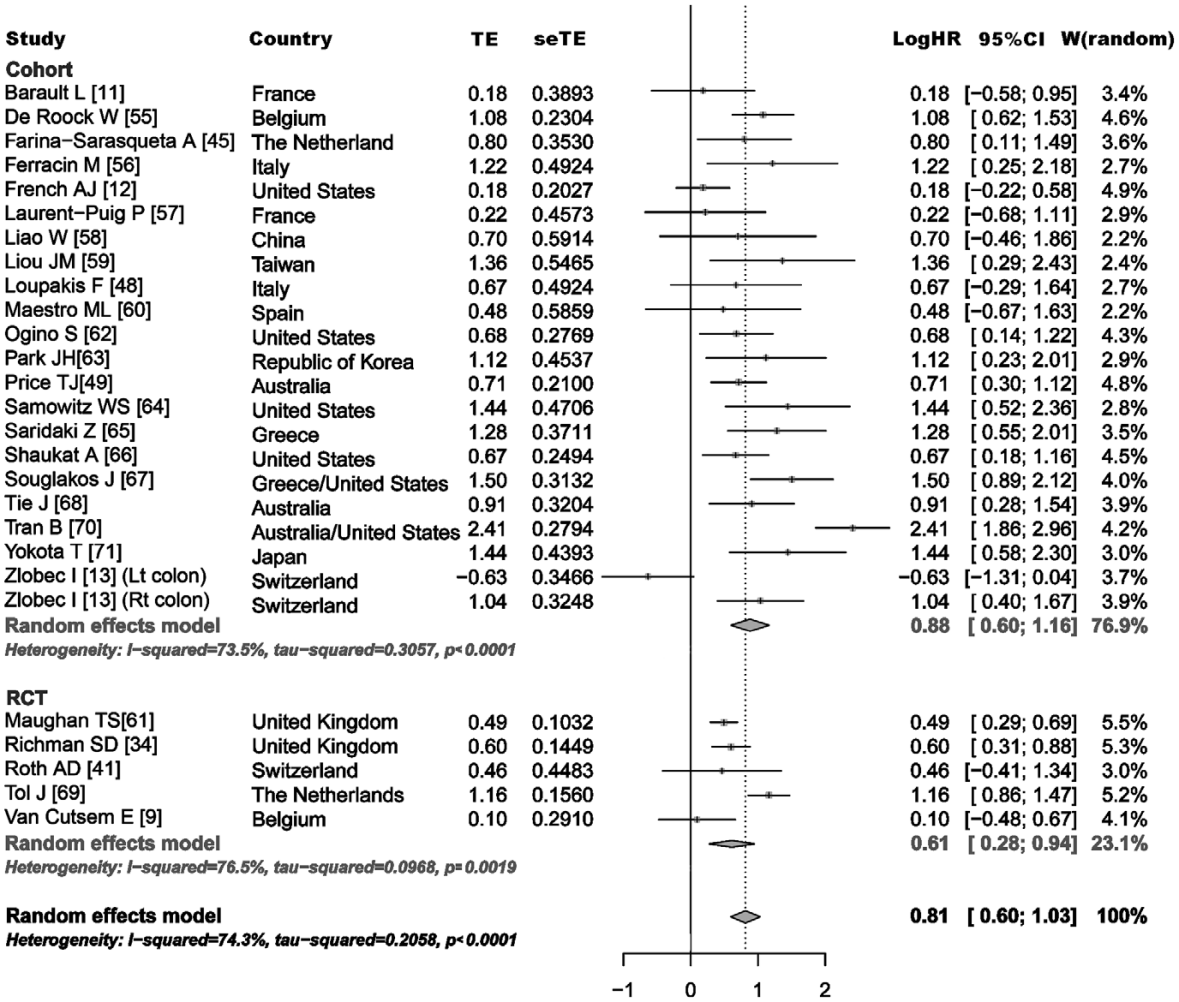
Random effect model of Log hazard ratio (LogHR) with 95% confidence interval for studies comparing the effect of *BRAF-*V600E mutation on overall survival of colorectal cancer patients. A LogHR <0 implies a survival benefit for patients with *BRAF* mutation. The square size indicates the power of each study in meta-analysis based on the number of patients in that study. The center of diamond shape at the lowest part indicates the combined LogHR for meta-analysis and its extremities the 95% confidence interval.

In our pooled data for colorectal cancer only one paper reported a protective HR (less than one) for *BRAF* mutation. Accordingly, Zlobec *et al* [13] observed a protective HR of 0.53 (0.3–1.3) for left side colon cancer. However, they reported a higher HR of 2.82 (1.5–5.5) for *BRAF* mutation as a risk factor for right side colon cancer in the same report. We considered these two analyses as separate reports in our final analysis. The pooled log HR of *BRAF* mutation effect on patient survival in colorectal cancer for cohort and RCT studies were 0.88 (0.60–1.16) and 0.61 (0.28–0.94), respectively. The final log HR for all studies on colorectal cancer was 0.81 (0.60–1.03) which corresponds to a HR of 2.24 (1.82–2.83, 95% CI). The heterogeneity of data on colorectal cancer was significant (*P*<0.0001) and I^2^ estimate of variation between analyzed studies was 74.3% (Figure 2).

### Impact of *BRAF-V600e* Mutation on Melanoma Patient Survival

**Figure 3:**
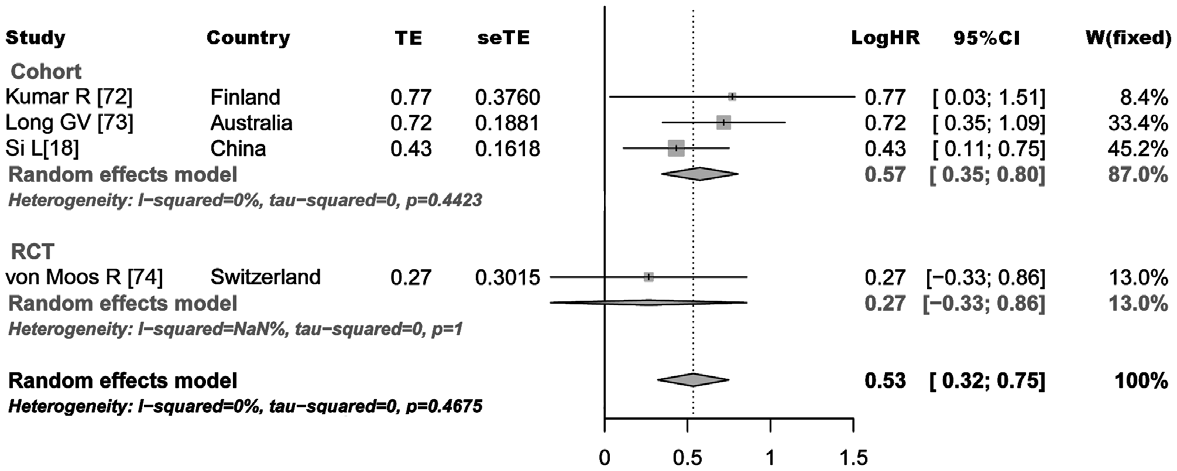
Random effect model of Log hazard ratio (LogHR) with 95% confidence interval for studies comparing the effect of *BRAF-*V600E mutation on overall survival in melanoma patients. A LogHR <0 implies a survival benefit for patients with *BRAF* mutation. The square size indicates the power of each study in meta-analysis based on the number of patients in that study. The center of diamond shape at the lowest part indicates the combined LogHR for meta-analysis and its extremities the 95% confidence interval.

One RCT study [21] compared *BRAF* mutation in patients’ serum level with tumor samples but had no data on wild type *BRAF* status. Two other RCTs evaluated progression-free survival (PFS) with either no overall survival information [22] and non-significant PFS or no overall survival data on wild type *BRAF* group [23]. One cohort study used age <55 years as a surrogate marker for *BRAF* mutation while others either reported PFS or non-significant difference with no detailed information or survival curve graphs (Table 1). Pooled log HR for *BRAF* mutation effect on patient survival in melanoma for cohort studies was 0.57 (0.35–0.80) and the final pooled log HR including one RCT was 0.53 (0.32–0.75) corresponding to a HR of 1.70 (1.37–2.12, 95% CI). The heterogeneity of the data was not significant (*P* = 0.467) and I^2^ estimate of variation between analyzed studies was 0.0% (Figure 3).

### Impact of *BRAF-V600e* Mutation on Papillary Thyroid Carcinoma Patient Survival

One study [24] reported no death in wild type *BRAF* group after almost 221 months of follow up. Another study [20] reported just one death in wild-type *BRAF* group after 20 years of follow up with odds ratio of 14.63 (1.28–167.29) for mutant *BRAF*. The study by Musholt *et al* [19] reported no difference in overall survival (HR = 1.04), while two other reports [10], [25] showed no difference in disease-free survival between mutant and wild-type *BRAF* patients. However, another study by Abubaker *et al* [26] found *BRAF* mutation as a risk factor for disease free survival and Costa *et al* [27] reported that *BRAF* mutation would affect patient survival only if it is considered in combination with other mutations but not alone. In addition, Wang *et al* [28] reported that patients with synchronous bilateral papillary thyroid carcinoma, which harbor more *BRAF* mutation, have worse survival compared with those who have unilateral papillary thyroid carcinoma (Table 2).

**Table 2:** Summary of studies that reported the status of *BRAF* mutation in papillary thyroid carcinoma with information on patient survival.

	Number of patients				Overall survival		Hazard ratio	Progression free survival		Hazard ratio
	Overall	*BRAF* subgroup	*BRAF* WT	*BRAF* mutant	*BRAF* mutant	*BRAF* WT		*BRAF* mutant	*BRAF* WT	
**Abubaker J ** [**26**]	536	296	143	153 (51.7%)				Poor		
**Costa AM ** [**27**]	49	49	22	27 (55%)	No diff.Poor, when combined with other markers					
**Elisei R ** [**20**]	102	102	64	38 (37.3%)	Sig. Lower		OR 14.63 (1.28–167.29)			
**Ito Y ** [**25**]	631	631	389	242 (38.4%)				DFSNo diff.		
**Musholt TJ ** [**19**]	290	290	168	122 (42%)	No diff.		1.04			
**Oler G ** [**75**]		120	62	57 (48%)				No data on survival		
**O’Neill CJ ** [**24**]	104	101	41	60 (59%)				80%	75%	
**Stanojevic B ** [**10**]		266	182	84 (31.6%)				DFS.No diff.		1.15(0.42–3.19)
**Wang W ** [**28**]	891177714	20867 (SBiPTC)141 (UiPTC)	932370	115(55.3%)44 (65.7%)71 (50.4%)	SBiPTC with more *BRAF* mutation had lower survival compared toUiPTC (*P* = 0.091)					
**Xing M ** [**76**]		219	112	107 (48.9%)	Recurrence free probability					

DFS, Disease free survival; OR, Odds Ratio; SBiPTC, Synchronous bilateral papillary thyroid carcinoma; UiPTC, Unilateral papillary thyroid carcinoma.

## Discussion

*BRAF* mutation has become an important research topic in cancer biology since the original observation by Davies *et al* [3] in 2002. They revealed that high frequency of *BRAF* mutation is a common phenomenon in multiple types of cancers. Since then, numerous studies investigated the role of *BRAF* mutation in cancer development and progression. In mechanistic point of view, *BRAF*-V600E mutation, as the most prevalent *BRAF* mutation, changes the inactive conformation of BRAF kinase to a very active state [6]. This simple point mutation leads to a constitutive activation of whole MAPK pathway, which mediates the cell surface growth signals to transcriptional activity of cell cycle regulatory genes. The key regulatory role of *BRAF* mutation in MAPK activation especially in melanoma generated a tremendous research effort to block this signaling pathway for cancer treatment. The usage of most available multi-kinase inhibitor at that time, sorafenib, was the first step toward targeted BRAF inhibition. Despite the first promising results in cell culture and animal studies, sorafenib was found to be unsuccessful in melanoma patients treatment even among those harboring mutant *BRAF* [29], [30]. A number of other small molecule inhibitors have been tested for targeted BRAF inhibition; however, so far only PLX4032 and GSK2118436 have successfully been used in clinical stages [31], [32]. Taking everything into account, the main goal in cancer treatment is to increase patient survival, while the idea of whether *BRAF* mutation per se actually affects patient survival has been a matter of debate. In this study, by conducting meta-analysis on data reported in 30 independent studies, we evaluated the effect of *BRAF*-V600E mutation on patient survival in colorectal cancer and melanoma. We also reviewed another 10 independent studies on papillary thyroid carcinoma in which *BRAF* mutation is prevalent.

In a population of 11,773 patients from 26 independent studies, we found that the risk of mortality in colorectal cancer patients harboring *BRAF*-V600E mutation is more than two times higher than those with wild-type *BRAF*. We also demonstrated that melanoma patients with *BRAF* mutation have a 1.7 times higher risk of mortality when compared with their counterparts without *BRAF* mutation in a population of 674 patients from the pooled result of 4 studies. In fact, this significant hazard ratio for *BRAF* mutation in our study can indirectly explain the previously reported promising improvement of melanoma patient survival harboring *BRAF* mutation after selective BRAF inhibitor treatments [32]–[34]. However, short period of symptom free survival and resistance to drug therapy are new emerging problems in BRAF specific inhibitor treatments in melanoma patients. Although the preliminary results for BRAF inhibitor treatments were promising, resistance to drug treatment usually appears in almost all cases [23], [35]. Typically a reactivation of MAPK pathway happens in resistant cases through other mechanisms including RAS or MEK1 mutations, COT overexpression or BRAF truncation [36]–[39]. Nevertheless, the response rate of colon cancer patients harboring *BRAF*-V600E mutation to BRAF inhibitor treatment is much lower than melanoma patients [40], [41]. In fact, over activation and crosstalk of parallel pathways like phosphatidylinositol 3-kinase (PI3 kinase) – AKT with MAPK in colorectal cancer is playing a main role in the observed different response to BRAF inhibitor treatments in colorectal cancer. Likewise, a very recent study by Prahallad *et al* [42] revealed the important role of epidermal growth factor receptor (EGFR) activation in colon cancer patients as well. They showed that a feedback activation of EGFR occurs in colon cancer cells after *BRAF*-V600E inhibition very quickly. In fact, this feedback activation of EGFR in colon cancer cells leads to a continuous malignant cell proliferation even in the presence of *BRAF*-V600E inhibition. However, this mechanism would not be applicable to melanoma cells as they express a very low level of EGFR [42].

*BRAF* mutation in papillary thyroid cancer was reported to be a risk factor for worse survival in two studies [20], [24]. Notwithstanding a notably long term follow-up of patients for 18 to 20 years in these studies from Australia and Italy, the authors either did not observe any death [24] or only one death [20] in *BRAF* wild-type group of patients. Authors reported only one death in a population of 64 or no death among 41 wild-type *BRAF* patients while Standardized Death Rate for general population in Australia was found to be 6.9 and 4.7 per 1000 standard populations for male and female respectively (http://www.abs.gov.au/ausstats/abs@.nsf/Lookup/by+Subject/4125.0∼Jul+2011∼Main+Features∼Death+rate∼3210). Also, based on the report from the Centers for Disease Control and Prevention, age specific mortality rate for normal population within the same age group as patients in these two studies (45 to 54 years) is 420.9 per 100,000 of population (http://www.cdc.gov/nchs/nvss/mortality_tables.htm). Altogether, it seems that more studies with larger sample size are required to determine the significance of *BRAF*-V600E mutation effect on papillary thyroid carcinoma patient survival.

The number of studies comparing molecular and clinicopathological difference between right and left side colon cancers have been increased during the past two decades. For instance, a higher frequency of microsatellite instability, which is a poor prognostic factor for colon cancer, has been reported to be more prevalent in right side compared with left side colon cancer [43], [44]. A number of studies also reported more prevalent *BRAF* mutations in right side colon cancer [13], [45]. Although different biological and clinicopathological characteristics have been described for right and left side colon cancer, this issue is still a matter of controversy. Accordingly, investigating a large number of patients (29,568) in a recent study, Benedix et al [46] revealed a remarkable clinicopathological variation among colonic subsites irrespective of the side of tumor (right versus left). They showed that these differences are more related to the anatomical site of the cancer origin rather than a simple right and left categorization [46], [47]. Despite a number of descriptive reports on the prevalence of *BRAF* mutation and its correlation with clinicopathological characteristics, there has been no comprehensive comparison on the effect of *BRAF* mutation on patient survival in separate groups of right and left side colon cancers. Accordingly, a controversial favorable effect for *BRAF* mutation on patient survival on left side colon cancer (*P* = 0.084) has been reported by Zlobec *et al* [13], while in the same study they observed a significant negative effect of *BRAF* mutation on patient survival for right side colon cancer (*P* = 0.01). They did observe a significant protective effect for *BRAF* mutation on left side colon cancer considering other risk factors in a multifactorial analysis (HR, 0.53; *P* = 0.109). However, the negative effect of *BRAF* mutation on right side colon cancer patient survival was persistently significant in multifactorial analysis (HR, 2.82; *P* = 0.002) [13]. A number of other researchers from our pooled studies also observed a considerable decrease in patient survival with *BRAF* mutation compared with wild-type *BRAF*; however, the specific HR for wild-type or mutant BRAF was not determined [9], [45], [48], [49]. Based on the significantly poor patient survival in mutant *BRAF* group in those studies according to survival curves and reported survival time difference, we estimated the HR of mutant *BRAF* in our meta-analysis.

With respect to reports on melanoma, Ellerhorst *et al* [50] reported no difference in patient survival between a group of patients with either *BRAF* mutation (109 cases) or *NRAS* mutation (31 cases) and wild-type *BRAF/NRAS* group (80 cases). There was no data available for the effect of *BRAF* mutation alone on patient survival in this report. In a very similar study, Houben *et al* [51] evaluated the effect of combined mutation of *BRAF* and *NRAS* mutation in 200 patients and reported a poor overall survival prognosis for metastatic samples which harbor either *BRAF* or *NRAS* mutation. However, they did not observe the same pattern in primary melanoma patients. As these two reports did not provide any information on the effect of *BRAF* mutation per se on patient survival we did not include them in our final meta-analysis. The inconsistency of results in these reports could be due to the fact that they combined *BRAF* and *NRAS* mutation and classified this group of patients together. In addition, Akslen *et al* [14] and Chang *et al* [15] reported no difference in patient survival in 69 and 68 cases respectively according to their *BRAF* mutation status. However, no details on patient survival have been provided in these reports. Akslen *et al* [14] mostly focused on different *BRAF* and *NRAS* mutations and their combinations and possible correlation with clinicopathologic characteristics. They reported that *BRAF* and *NRAS* mutations are mutually exclusive except for one case but they did not find any correlation with tumor cell proliferation, thickness or vascular invasion. Although they reported a median follow-up time of 76 months for the patients, no detailed information on mean survival time in each arm of the study was provided. There was no survival curve available in this report either. In a separate study, Chang *et al* [15] observed a significant trend for liver metastasis and tendency for multiple organ metastasis in *BRAF* mutant group but they did not detect a significant difference in either clinicopathological characteristics or in patient survival. Basically in this study authors chose a descriptive method to explain their observation and just mentioned that they did not find any correlation between patient survival and *BRAF* mutation. Unfortunately, no more detailed information including mean survival time in each group of study or a survival graph has been provided by the authors. A need for a conclusive meta-analysis on the effect of *BRAF* mutation on melanoma patient survival has been emerged due to the controversial reports on this issue. In our meta-analysis, we combined the results of four independent studies and measured the pooled risk of *BRAF* mutation on melanoma patient survival. So far our report is the first study on this issue which demonstrates the correlation between *BRAF* mutation and poor melanoma patient survival in a reliable statistical point of view. The number of reports on *BRAF* mutation and colorectal cancer were enough to pool the results together and perform a meta-analysis. Therefore, our findings in the pooled data suggest that with successful BRAF inhibition we would be able to increase the survival of colorectal cancer and melanoma patients harboring *BRAF* mutation.

BRAF plays a very important role in cancer initiation and progression. Mutation of *BRAF* is detected in all stages of melanocytic lesions including nevi, primary and metastatic melanoma. It is known to be involved in the multiple stages of tumor progression such as cell proliferation [52] and invasion [8]. Interestingly, BRAF has also been shown to be involved in the progression of melanoma toward metastasis by enhancing its migration [53]. However, cancer is a complex disease with multiple markers being involved in its formation and progression. Therefore, simultaneous study of other factors involved in BRAF network is crucial for a better understanding of its role in cancer. For instance, the cooperation between *BRAF* mutation and PTEN loss in melanoma progression has been identified [54]. Since improving patient survival is the main goal in cancer treatment, further meta-analysis evaluation on the combination of markers involved in this critical network including *RAS* and *PTEN* with *BRAF* seems necessary for future planning in cancer treatment and drug development.

In summary, we used systematic review and meta-analysis approach to investigate possible association between *BRAF*-V600E mutation and cancer patient survival. We found that *BRAF*-V600E mutation increases the risk of mortality in colorectal cancer patients for more than two-fold. In addition, we revealed that *BRAF*-V600E mutation also significantly increases the risk of mortality in melanoma patients. This data highlights the important role of mutant *BRAF* in patient survival and suggest that with successful BRAF inhibition we may be able to increase the survival of colorectal cancer and melanoma patients harboring *BRAF* mutation.

## Supporting Information

Figure S1 Complete PRISMA search for Pubmed and EMBASE 2002–2011.
(DOC)

Table S1 PRISMA checklist.
(DOC)

## References

[1] Kyriakis JM, App H, Zhang XF, Banerjee P, Brautigan DL, et al (1992) Raf-1 activates MAP kinase-kinase. Nature 358: 417–421. 1322500 10.1038/358417a0

[2] Malumbres M, Barbacid M (2003) RAS oncogenes: the first 30 years. Nat Rev Cancer 3: 459–465. 12778136 10.1038/nrc1097

[3] Davies H, Bignell GR, Cox C, Stephens P, Edkins S, et al (2002) Mutations of the BRAF gene in human cancer. Nature 417: 949–954. 12068308 10.1038/nature00766

[4] Brose MS, Volpe P, Feldman M, Kumar M, Rishi I, et al (2002) BRAF and RAS mutations in human lung cancer and melanoma. Cancer Res 62: 6997–7000. 12460918

[5] Cohen Y, Xing M, Mambo E, Guo Z, Wu G, et al (2003) BRAF mutation in papillary thyroid carcinoma. J Natl Cancer Inst 95: 625–627. 12697856 10.1093/jnci/95.8.625

[6] Wan PT, Garnett MJ, Roe SM, Lee S, Niculescu-Duvaz D, et al (2004) Mechanism of activation of the RAF-ERK signaling pathway by oncogenic mutations of B-RAF. Cell 116: 855–867. 15035987 10.1016/s0092-8674(04)00215-6

[7] Wellbrock C, Ogilvie L, Hedley D, Karasarides M, Martin J, et al (2004) V599EB-RAF is an oncogene in melanocytes. Cancer Res 64: 2338–2342. 15059882 10.1158/0008-5472.can-03-3433

[8] Hoeflich KP, Gray DC, Eby MT, Tien JY, Wong L, et al (2006) Oncogenic BRAF is required for tumor growth and maintenance in melanoma models. Cancer Res 66: 999–1006. 16424035 10.1158/0008-5472.CAN-05-2720

[9] Van Cutsem E, Kohne CH, Lang I, Folprecht G, Nowacki MP, et al (2011) Cetuximab plus irinotecan, fluorouracil, and leucovorin as first-line treatment for metastatic colorectal cancer: updated analysis of overall survival according to tumor KRAS and BRAF mutation status. J Clin Oncol 29: 2011–2019. 21502544 10.1200/JCO.2010.33.5091

[10] Stanojevic B, Dzodic R, Saenko V, Milovanovic Z, Pupic G, et al (2011) Mutational and clinico-pathological analysis of papillary thyroid carcinoma in Serbia. Endocr J 58: 381–393. 21498916 10.1507/endocrj.k11e-054

[11] Barault L, Charon-Barra C, Jooste V, de la Vega MF, Martin L, et al (2008) Hypermethylator phenotype in sporadic colon cancer: study on a population-based series of 582 cases. Cancer Res 68: 8541–8546. 18922929 10.1158/0008-5472.CAN-08-1171

[12] French AJ, Sargent DJ, Burgart LJ, Foster NR, Kabat BF, et al (2008) Prognostic significance of defective mismatch repair and BRAF V600E in patients with colon cancer. Clin Cancer Res 14: 3408–3415. 18519771 10.1158/1078-0432.CCR-07-1489 PMC2674786

[13] Zlobec I, Bihl MP, Schwarb H, Terracciano L, Lugli A (2010) Clinicopathological and protein characterization of BRAF- and K-RAS-mutated colorectal cancer and implications for prognosis. Int J Cancer 127: 367–380. 19908233 10.1002/ijc.25042

[14] Akslen LA, Angelini S, Straume O, Bachmann IM, Molven A, et al (2005) BRAF and NRAS mutations are frequent in nodular melanoma but are not associated with tumor cell proliferation or patient survival. J Invest Dermatol 125: 312–317. 16098042 10.1111/j.0022-202X.2005.23788.x

[15] Chang DZ, Panageas KS, Osman I, Polsky D, Busam K, et al (2004) Clinical significance of BRAF mutations in metastatic melanoma. J Transl Med 2: 46. 15613230 10.1186/1479-5876-2-46 PMC544849

[16] Greenland S (1998) Meta-analysis. In: Rothman KJ, Greenland, S., editor. Modern epidemiology. 2nd ed. Philadelphia: Lippincott-Raven. 643–673.

[17] Parmar MK, Torri V, Stewart L (1998) Extracting summary statistics to perform meta-analyses of the published literature for survival endpoints. Stat Med 17: 2815–2834. 9921604 10.1002/(sici)1097-0258(19981230)17:24<2815::aid-sim110>3.0.co;2-8

[18] Si L, Kong Y, Xu X, Flaherty KT, Sheng X, et al (2012) Prevalence of BRAF V600E mutation in Chinese melanoma patients: large scale analysis of BRAF and NRAS mutations in a 432-case cohort. Eur J Cancer 48: 94–100. 21788131 10.1016/j.ejca.2011.06.056

[19] Musholt TJ, Schonefeld S, Schwarz CH, Watzka FM, Musholt PB, et al (2010) Impact of pathognomonic genetic alterations on the prognosis of papillary thyroid carcinoma. ESES vienna presentation. Langenbecks Arch Surg 395: 877–883. 20640859 10.1007/s00423-010-0682-6

[20] Elisei R, Ugolini C, Viola D, Lupi C, Biagini A, et al (2008) BRAF(V600E) mutation and outcome of patients with papillary thyroid carcinoma: a 15-year median follow-up study. J Clin Endocrinol Metab 93: 3943–3949. 18682506 10.1210/jc.2008-0607

[21] Board RE, Ellison G, Orr MC, Kemsley KR, McWalter G, et al (2009) Detection of BRAF mutations in the tumour and serum of patients enrolled in the AZD6244 (ARRY-142886) advanced melanoma phase II study. Br J Cancer 101: 1724–1730. 19861964 10.1038/sj.bjc.6605371 PMC2778539

[22] Amaravadi RK, Schuchter LM, McDermott DF, Kramer A, Giles L, et al (2009) Phase II Trial of Temozolomide and Sorafenib in Advanced Melanoma Patients with or without Brain Metastases. Clin Cancer Res 15: 7711–7718. 19996224 10.1158/1078-0432.CCR-09-2074 PMC2795076

[23] Flaherty KT, Puzanov I, Kim KB, Ribas A, McArthur GA, et al (2010) Inhibition of mutated, activated BRAF in metastatic melanoma. N Engl J Med 363: 809–819. 20818844 10.1056/NEJMoa1002011 PMC3724529

[24] O'Neill CJ, Bullock M, Chou A, Sidhu SB, Delbridge LW, et al.. (2010) BRAF(V600E) mutation is associated with an increased risk of nodal recurrence requiring reoperative surgery in patients with papillary thyroid cancer. Surgery 148: 1139–1145; discussion 1145–1136. 10.1016/j.surg.2010.09.005 21134544

[25] Ito Y, Yoshida H, Maruo R, Morita S, Takano T, et al (2009) BRAF mutation in papillary thyroid carcinoma in a Japanese population: its lack of correlation with high-risk clinicopathological features and disease-free survival of patients. Endocr J 56: 89–97. 18840924 10.1507/endocrj.k08e-208

[26] Abubaker J, Jehan Z, Bavi P, Sultana M, Al-Harbi S, et al (2008) Clinicopathological analysis of papillary thyroid cancer with PIK3CA alterations in a Middle Eastern population. J Clin Endocrinol Metab 93: 611–618. 18000091 10.1210/jc.2007-1717

[27] Costa AM, Herrero A, Fresno MF, Heymann J, Alvarez JA, et al (2008) BRAF mutation associated with other genetic events identifies a subset of aggressive papillary thyroid carcinoma. Clin Endocrinol (Oxf) 68: 618–634. 18070147 10.1111/j.1365-2265.2007.03077.x

[28] Wang W, Zhao W, Wang H, Teng X, Chen X, et al (2012) Poorer Prognosis and Higher Prevalence of BRAF (V600E) Mutation in Synchronous Bilateral Papillary Thyroid Carcinoma. Ann Surg Oncol 19: 31–36. 22033631 10.1245/s10434-011-2096-2

[29] Sharma A, Trivedi NR, Zimmerman MA, Tuveson DA, Smith CD, et al (2005) Mutant V599EB-Raf regulates growth and vascular development of malignant melanoma tumors. Cancer Res 65: 2412–2421. 15781657 10.1158/0008-5472.CAN-04-2423

[30] Hauschild A, Agarwala SS, Trefzer U, Hogg D, Robert C, et al (2009) Results of a phase III, randomized, placebo-controlled study of sorafenib in combination with carboplatin and paclitaxel as second-line treatment in patients with unresectable stage III or stage IV melanoma. J Clin Oncol 27: 2823–2830. 19349552 10.1200/JCO.2007.15.7636

[31] Bollag G, Hirth P, Tsai J, Zhang J, Ibrahim PN, et al (2010) Clinical efficacy of a RAF inhibitor needs broad target blockade in BRAF-mutant melanoma. Nature 467: 596–599. 20823850 10.1038/nature09454 PMC2948082

[32] Hauschild A, Grob JJ, Demidov LV, Jouary T, Gutzmer R, et al (2012) Dabrafenib in BRAF-mutated metastatic melanoma: a multicentre, open-label, phase 3 randomised controlled trial. Lancet 380: 358–365. 22735384 10.1016/S0140-6736(12)60868-X

[33] Young K, Minchom A, Larkin J (2012) BRIM-1, -2 and -3 trials: improved survival with vemurafenib in metastatic melanoma patients with a BRAF(V600E) mutation. Future Oncol 8: 499–507. 22646765 10.2217/fon.12.43

[34] Richman SD, Seymour MT, Chambers P, Elliott F, Daly CL, et al (2009) KRAS and BRAF mutations in advanced colorectal cancer are associated with poor prognosis but do not preclude benefit from oxaliplatin or irinotecan: results from the MRC FOCUS trial. J Clin Oncol 27: 5931–5937. 19884549 10.1200/JCO.2009.22.4295

[35] Chapman PB, Hauschild A, Robert C, Haanen JB, Ascierto P, et al (2011) Improved survival with vemurafenib in melanoma with BRAF V600E mutation. N Engl J Med 364: 2507–2516. 21639808 10.1056/NEJMoa1103782 PMC3549296

[36] Paraiso KH, Fedorenko IV, Cantini LP, Munko AC, Hall M, et al (2010) Recovery of phospho-ERK activity allows melanoma cells to escape from BRAF inhibitor therapy. Br J Cancer 102: 1724–1730. 20531415 10.1038/sj.bjc.6605714 PMC2883709

[37] Johannessen CM, Boehm JS, Kim SY, Thomas SR, Wardwell L, et al (2010) COT drives resistance to RAF inhibition through MAP kinase pathway reactivation. Nature 468: 968–972. 21107320 10.1038/nature09627 PMC3058384

[38] Poulikakos PI, Persaud Y, Janakiraman M, Kong X, Ng C, et al (2011) RAF inhibitor resistance is mediated by dimerization of aberrantly spliced BRAF(V600E). Nature 480: 387–390. 22113612 10.1038/nature10662 PMC3266695

[39] Wagle N, Emery C, Berger MF, Davis MJ, Sawyer A, et al (2011) Dissecting therapeutic resistance to RAF inhibition in melanoma by tumor genomic profiling. J Clin Oncol 29: 3085–3096. 21383288 10.1200/JCO.2010.33.2312 PMC3157968

[40] Kopetz S, Desai J, Chan E, Hecht JR, O'Dwyer PJ, et al.. (2010) PLX4032 in metastatic colon cancer patients with mutant BRAF tumors. J Clin Oncol 28: abstract 3534.

[41] Roth AD, Tejpar S, Delorenzi M, Yan P, Fiocca R, et al (2010) Prognostic role of KRAS and BRAF in stage II and III resected colon cancer: results of the translational study on the PETACC-3, EORTC 40993, SAKK 60-00 trial. J Clin Oncol 28: 466–474. 20008640 10.1200/JCO.2009.23.3452

[42] Prahallad A, Sun C, Huang S, Di Nicolantonio F, Salazar R, et al (2012) Unresponsiveness of colon cancer to BRAF(V600E) inhibition through feedback activation of EGFR. Nature 483: 100–103. 22281684 10.1038/nature10868

[43] Nash GM, Gimbel M, Cohen AM, Zeng ZS, Ndubuisi MI, et al (2010) KRAS mutation and microsatellite instability: two genetic markers of early tumor development that influence the prognosis of colorectal cancer. Ann Surg Oncol 17: 416–424. 19813061 10.1245/s10434-009-0713-0 PMC4380015

[44] Rampazzo E, Bertorelle R, Serra L, Terrin L, Candiotto C, et al (2010) Relationship between telomere shortening, genetic instability, and site of tumour origin in colorectal cancers. Br J Cancer 102: 1300–1305. 20386541 10.1038/sj.bjc.6605644 PMC2856015

[45] Farina-Sarasqueta A, van Lijnschoten G, Moerland E, Creemers GJ, Lemmens VE, et al (2010) The BRAF V600E mutation is an independent prognostic factor for survival in stage II and stage III colon cancer patients. Ann Oncol 21: 2396–2402. 20501503 10.1093/annonc/mdq258

[46] Benedix F, Schmidt U, Mroczkowski P, Gastinger I, Lippert H, et al (2011) Colon carcinoma-classification into right and left sided cancer or according to colonic subsite?-Analysis of 29,568 patients. Eur J Surg Oncol 37: 134–139. 21193285 10.1016/j.ejso.2010.12.004

[47] Benedix F, Meyer F, Kube R, Kropf S, Kuester D, et al.. (2012) Influence of anatomical subsite on the incidence of microsatellite instability, and KRAS and BRAF mutation rates in patients with colon carcinoma. Pathol Res Pract (in press). 10.1016/j.prp.2012.07.003 22898351

[48] Loupakis F, Ruzzo A, Cremolini C, Vincenzi B, Salvatore L, et al (2009) KRAS codon 61, 146 and BRAF mutations predict resistance to cetuximab plus irinotecan in KRAS codon 12 and 13 wild-type metastatic colorectal cancer. Br J Cancer 101: 715–721. 19603018 10.1038/sj.bjc.6605177 PMC2736831

[49] Price TJ, Hardingham JE, Lee CK, Weickhardt A, Townsend AR, et al (2011) Impact of KRAS and BRAF Gene Mutation Status on Outcomes From the Phase III AGITG MAX Trial of Capecitabine Alone or in Combination With Bevacizumab and Mitomycin in Advanced Colorectal Cancer. J Clin Oncol 29: 2675–2682. 21646616 10.1200/JCO.2010.34.5520

[50] Ellerhorst JA, Greene VR, Ekmekcioglu S, Warneke CL, Johnson MM, et al (2011) Clinical correlates of NRAS and BRAF mutations in primary human melanoma. Clin Cancer Res 17: 229–235. 20975100 10.1158/1078-0432.CCR-10-2276 PMC3022950

[51] Houben R, Becker JC, Kappel A, Terheyden P, Brocker EB, et al (2004) Constitutive activation of the Ras-Raf signaling pathway in metastatic melanoma is associated with poor prognosis. J Carcinog 3: 6. 15046639 10.1186/1477-3163-3-6 PMC420489

[52] Kumar R, Angelini S, Snellman E, Hemminki K (2004) BRAF mutations are common somatic events in melanocytic nevi. J Invest Dermatol 122: 342–348. 15009715 10.1046/j.0022-202X.2004.22225.x

[53] Arozarena I, Sanchez-Laorden B, Packer L, Hidalgo-Carcedo C, Hayward R, et al (2011) Oncogenic BRAF induces melanoma cell invasion by downregulating the cGMP-specific phosphodiesterase PDE5A. Cancer Cell 19: 45–57. 21215707 10.1016/j.ccr.2010.10.029

[54] Dankort D, Curley DP, Cartlidge RA, Nelson B, Karnezis AN, et al (2009) Braf(V600E) cooperates with Pten loss to induce metastatic melanoma. Nat Genet 41: 544–552. 19282848 10.1038/ng.356 PMC2705918

[55] De Roock W, Claes B, Bernasconi D, De Schutter J, Biesmans B, et al (2010) Effects of KRAS, BRAF, NRAS, and PIK3CA mutations on the efficacy of cetuximab plus chemotherapy in chemotherapy-refractory metastatic colorectal cancer: a retrospective consortium analysis. Lancet Oncol 11: 753–762. 20619739 10.1016/S1470-2045(10)70130-3

[56] Ferracin M, Gafa R, Miotto E, Veronese A, Pultrone C, et al (2008) The methylator phenotype in microsatellite stable colorectal cancers is characterized by a distinct gene expression profile. J Pathol 214: 594–602. 18241079 10.1002/path.2318

[57] Laurent-Puig P, Cayre A, Manceau G, Buc E, Bachet JB, et al (2009) Analysis of PTEN, BRAF, and EGFR status in determining benefit from cetuximab therapy in wild-type KRAS metastatic colon cancer. J Clin Oncol 27: 5924–5930. 19884556 10.1200/JCO.2008.21.6796

[58] Liao W, Liao Y, Zhou JX, Xie J, Chen J, et al (2010) Gene mutations in epidermal growth factor receptor signaling network and their association with survival in Chinese patients with metastatic colorectal cancers. Anat Rec (Hoboken) 293: 1506–1511. 20652941 10.1002/ar.21202

[59] Liou JM, Wu MS, Shun CT, Chiu HM, Chen MJ, et al (2011) Mutations in BRAF correlate with poor survival of colorectal cancers in Chinese population. Int J Colorectal Dis 26: 1387–1395. 21553007 10.1007/s00384-011-1229-1

[60] Maestro ML, Vidaurreta M, Sanz-Casla MT, Rafael S, Veganzones S, et al (2007) Role of the BRAF mutations in the microsatellite instability genetic pathway in sporadic colorectal cancer. Ann Surg Oncol 14: 1229–1236. 17195912 10.1245/s10434-006-9111-z

[61] Maughan TS, Adams RA, Smith CG, Meade AM, Seymour MT, et al (2011) Addition of cetuximab to oxaliplatin-based first-line combination chemotherapy for treatment of advanced colorectal cancer: results of the randomised phase 3 MRC COIN trial. Lancet 377: 2103–2114. 21641636 10.1016/S0140-6736(11)60613-2 PMC3159415

[62] Ogino S, Nosho K, Kirkner GJ, Kawasaki T, Meyerhardt JA, et al (2009) CpG island methylator phenotype, microsatellite instability, BRAF mutation and clinical outcome in colon cancer. Gut 58: 90–96. 18832519 10.1136/gut.2008.155473 PMC2679586

[63] Park JH, Han SW, Oh DY, Im SA, Jeong SY, et al (2011) Analysis of KRAS, BRAF, PTEN, IGF1R, EGFR intron 1 CA status in both primary tumors and paired metastases in determining benefit from cetuximab therapy in colon cancer. Cancer Chemother Pharmacol 68: 1045–1055. 21340604 10.1007/s00280-011-1586-z

[64] Samowitz WS, Sweeney C, Herrick J, Albertsen H, Levin TR, et al (2005) Poor survival associated with the BRAF V600E mutation in microsatellite-stable colon cancers. Cancer Res 65: 6063–6069. 16024606 10.1158/0008-5472.CAN-05-0404

[65] Saridaki Z, Tzardi M, Papadaki C, Sfakianaki M, Pega F, et al (2011) Impact of KRAS, BRAF, PIK3CA mutations, PTEN, AREG, EREG expression and skin rash in >/ = 2 line cetuximab-based therapy of colorectal cancer patients. PLoS One 6: e15980. 21283802 10.1371/journal.pone.0015980 PMC3024325

[66] Shaukat A, Arain M, Thaygarajan B, Bond JH, Sawhney M (2010) Is BRAF mutation associated with interval colorectal cancers? Dig Dis Sci 55: 2352–2356. 20300843 10.1007/s10620-010-1182-9

[67] Souglakos J, Philips J, Wang R, Marwah S, Silver M, et al (2009) Prognostic and predictive value of common mutations for treatment response and survival in patients with metastatic colorectal cancer. Br J Cancer 101: 465–472. 19603024 10.1038/sj.bjc.6605164 PMC2720232

[68] Tie J, Gibbs P, Lipton L, Christie M, Jorissen RN, et al (2011) Optimizing targeted therapeutic development: analysis of a colorectal cancer patient population with the BRAF(V600E) mutation. Int J Cancer 128: 2075–2084. 20635392 10.1002/ijc.25555

[69] Tol J, Dijkstra JR, Klomp M, Teerenstra S, Dommerholt M, et al (2010) Markers for EGFR pathway activation as predictor of outcome in metastatic colorectal cancer patients treated with or without cetuximab. Eur J Cancer 46: 1997–2009. 20413299 10.1016/j.ejca.2010.03.036

[70] Tran B, Kopetz S, Tie J, Gibbs P, Jiang ZQ, et al.. (2012) Impact of BRAF mutation and microsatellite instability on the pattern of metastatic spread and prognosis in metastatic colorectal cancer. Cancer (in press). 10.1002/cncr.26086 PMC4257471 21456008

[71] Yokota T, Ura T, Shibata N, Takahari D, Shitara K, et al (2011) BRAF mutation is a powerful prognostic factor in advanced and recurrent colorectal cancer. Br J Cancer 104: 856–862. 21285991 10.1038/bjc.2011.19 PMC3048210

[72] Kumar R, Angelini S, Czene K, Sauroja I, Hahka-Kemppinen M, et al (2003) BRAF mutations in metastatic melanoma: a possible association with clinical outcome. Clin Cancer Res 9: 3362–3368. 12960123

[73] Long GV, Menzies AM, Nagrial AM, Haydu LE, Hamilton AL, et al (2011) Prognostic and clinicopathologic associations of oncogenic BRAF in metastatic melanoma. J Clin Oncol 29: 1239–1246. 21343559 10.1200/JCO.2010.32.4327

[74] von Moos R, Seifert B, Simcock M, Goldinger SM, Gillessen S, et al (2012) First-line temozolomide combined with bevacizumab in metastatic melanoma: a multicentre phase II trial (SAKK 50/07). Ann Oncol 23: 531–536. 21527587 10.1093/annonc/mdr126

[75] Oler G, Cerutti JM (2009) High prevalence of BRAF mutation in a Brazilian cohort of patients with sporadic papillary thyroid carcinomas: correlation with more aggressive phenotype and decreased expression of iodide-metabolizing genes. Cancer 115: 972–980. 19152441 10.1002/cncr.24118

[76] Xing M, Westra WH, Tufano RP, Cohen Y, Rosenbaum E, et al (2005) BRAF mutation predicts a poorer clinical prognosis for papillary thyroid cancer. J Clin Endocrinol Metab 90: 6373–6379. 16174717 10.1210/jc.2005-0987

