# Supplementary material for: The Prognostic Value of BRAF Mutation in Colorectal Cancer and Melanoma: A Systematic Review and Meta-Analysis
Source: PLoS One. 2012 Oct 9;7(10):e47054. doi: 10.1371/journal.pone.0047054 (PMC3467229; doi:10.1371/journal.pone.0047054)
Supplement: Figure S1 — Complete PRISMA search for Pubmed and EMBASE 2002–2011. (DOC) [file pone.0047054.s001.doc]

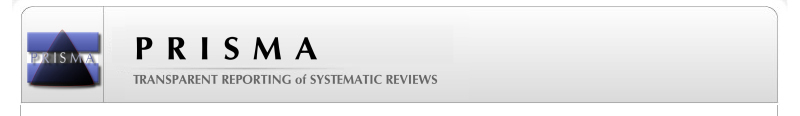
**Supporting Information Figure S1. PRISMA 2009 Flow Diagram**

**Screening**

**Included**

**Eligibility**

**Identification**

Records identified through database searching
(n = 565)

Additional records identified through other sources
(n = 7)

Records after duplicates removed
(n = 572)

Records screened
(n = 565)

Records excluded
(n = 445)

Full-text articles assessed for eligibility
(n = 127)

Full-text articles excluded, with reasons
(n = 87)

Studies included in qualitative synthesis
(n = 10)

Studies included in quantitative synthesis (meta-analysis)
(n = 30)
